# Focal Epithelial Hyperplasia (Heck’s Disease) in a 57-Year-Old Brazilian Patient: A Case Report and Literature Review

**DOI:** 10.14740/jocmr2466w

**Published:** 2016-02-27

**Authors:** Luciano Alberto de Castro, Joao Gabriel Leite de Castro, Alexandre Duarte Lopes da Cruz, Bruno Henrique de Sousa Barbosa, Jose Vieira de Spindula-Filho, Mauricio Barcelos Costa

**Affiliations:** a School of Medicine, Federal University of Tocantins, Palmas-TO, Brazil; b Department of Biological and Biomedical Sciences, Pontifical Catholic University of Goias, Goiania-GO, Brazil; c Department of Pathology and Laboratory Medicine, School of Medicine, Federal University of Goias, Goiania-GO, Brazil

**Keywords:** Focal epithelial hyperplasia, Heck’s disease, Human papillomavirus, Elderly patients, Biopsy

## Abstract

Focal epithelial hyperplasia (FEH), or Heck’s disease, is a rare disease of the oral mucosa associated with infection by some subtypes of human papilloma virus, especially subtypes 13 or 32. The disease is predominantly found in children and adolescents with indigenous heritage, but other ethnic groups can be affected worldwide. To the best of the authors’ knowledge, it has not been reported in Brazil’s elderly population. This article describes a case of FEH in a 57-year-old Brazilian patient presenting since childhood, with multiple lesions in the lips, buccal mucosa and tongue. The solitary tongue lesion underwent excisional biopsy and the histopathological analysis showed parakeratosis, acanthosis, rete pegs with a club-shaped appearance, koilocytosis and the presence of mitosoid cells. These microscopic findings in conjunction with clinical presentation were sufficient to establish the accurate diagnosis of FEH. Polymerase chain reaction (PCR) was performed, but no one human papillomavirus (HPV) subtype could be identified. Clinicians must be aware of this rare oral disease, which can even affect elderly patients, as we described here. Treatment may be indicated in selected cases due to esthetic and/or functional problems.

## Introduction

Focal epithelial hyperplasia (FEH), also known as Heck’s disease, is a rare benign disease first described in the English literature by Archard et al (1965) as multiple oral lesions affecting children belonging to the Navajo and other Native American tribes [1]. In the same year, Witkop and Niswander reported additional cases affecting Indians residing in Central and South America [2]. Furthermore, there were reports of the disease in other ethnic groups and individual cases worldwide [3-7].

The concentration of FEH cases in some geographical areas with familial occurrence suggests environmental influences and genetic predisposition in its etiology. However, Heck’s disease is primarily considered an infectious illness caused by human papillomavirus (HPV), especially 13 and 32 subtypes [3, 7, 8].

FEH is predominantly found in children and adolescents with indigenous heritage and has a variable female predilection [9-11]. Clinically, the disease is characterized by presence of multiple, soft, sessile papules and nodules, measuring 1 - 10 mm in diameter, presenting a color similar to circumjacent mucosa and usually affecting lips, buccal mucosa and the tongue [8, 12].

The diagnosis is based on clinical grounds, and treatment is usually unnecessary since most of lesions regress spontaneously and there is no tendency to malignant transformation [8, 12]. Therefore, the management of FEH lesions is only required for esthetic or functional purposes [6, 8, 12]. This paper reports an unusual case of FEH affecting an elderly patient who underwent excisional biopsy of a tongue lesion for functional reasons.

## Case Report

A brown-skinned 57-year-old Brazilian male complaining of numerous lumps in his upper lip and tongue was referred to the oral medicine center at the municipal health service of Palmas-Tocantins, Brazil. The patient reported that he had noticed such lesions since childhood and that they were painless. However, the lingual lump was often traumatized during mastication, causing bleeding and difficulty in feeding. His past medical history was non-contributory and the patient denied the presence of similar lesions in other family members.

**Figure 1:**
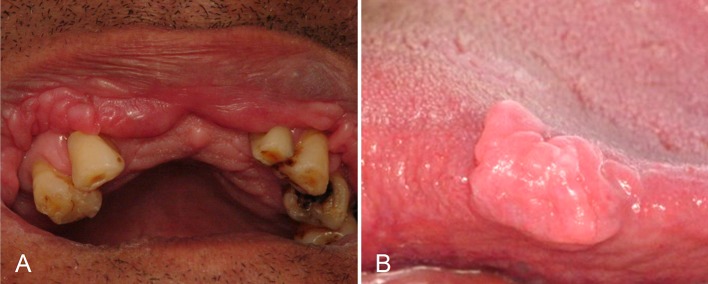
Multiple papules and nodules, sessile, coalescent, normal in colour, located on the upper lip mucosa and causing double lip appearance (A). Solitary tongue nodule, sessile, measuring approximately 20 mm on its largest diameter, with smooth and lobulated surface, similar in colour to the surrounding mucosa (B).

Extraoral examination did not show abnormalities. Intraoral examination revealed multiple lip lesions presenting themselves as normochromic, sessile and scattered papules and nodules, with a tendency to coalesce into plaques yielding double lip appearance (Fig. 1A). On the right tongue border, a large solitary nodule could be seen, measuring approximately 20 mm on its largest diameter, with a sessile, smooth and lobed surface and similar in color to the adjacent mucosa (Fig. 1B).

**Figure 2:**
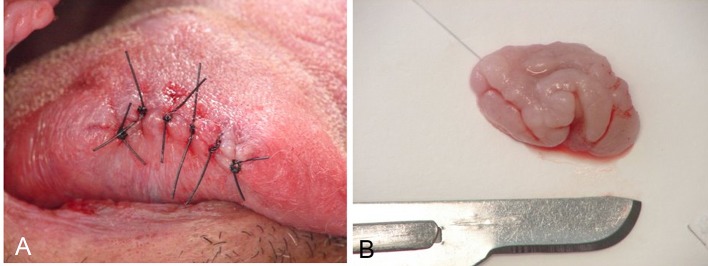
Immediate postoperative aspect (A) and surgical specimen (B).

The clinical diagnosis was FEH. The patient denied indigenous descent, although he was born in the northern region of Brazil, in which there are many Indian tribes. With the purpose of diagnostic confirmation and elimination of chewing discomfort, the excisional biopsy of the tongue nodule was performed under local anesthesia. There were no complications during the surgery nor during the postoperative period (Fig. 2).

**Figure 3:**
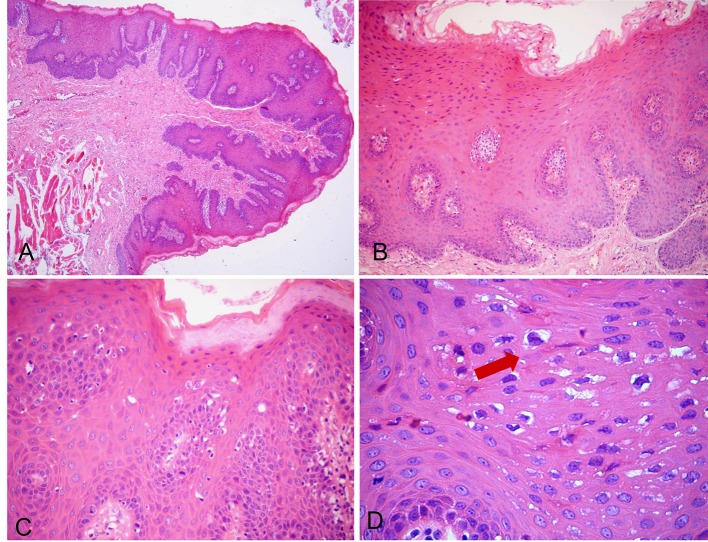
H&E-stained photomicrographs displaying characteristic features of FEH. Low-power magnification shows pseudocarcinomatous hyperplasia, marked acanthosis and elongated rete ridges (A, × 40; B, × 100). Spinous layer with parakeratosis, anisokaryosis and typical koilocytosis (C, × 200). High magnification allows clearer depiction of koilocytosis showing keratinocytes with pyknotic nuclei, surrounded by clears areas (D, × 400, arrow).

Histopathological analysis showed stratified squamous epithelium with parakeratosis, acanthosis and horizontal fusion of elongated epithelial rete ridges. Some rete pegs got a club-shaped appearance. The spinous layer exhibited individual keratinocytes with nuclear fragmentation resembling a mitotic figure (mitosoid cells) and other groups of cells with pycnotic or absent nuclei and clear cytoplasms (koilocytes). The underlying connective tissue consisted of abundant and wavy collagen bundles interspersed with fibroblast nuclei and blood vessels, some of which congested. Scarce inflammatory cells could be seen in the subepithelial area (Fig. 3). These data confirmed the clinical hypothesis of FEH.

In other examinations to identify the presumed HPV subtypes in the specimen, paraffin-embedded block was submitted to polymerase chain reaction (PCR) analysis. First, DNA was isolated from the 5 μm sections of formalin-fixed, paraffin-embedded tissue specimens. The samples were deparaffinised in microcentrifuge tubes, then digested by 400 mg/mL proteinase-K in 200 mL TE10E1 buffer at 55 °C for 24 - 48 h. After heat inactivation of the enzyme, DNA was isolated by using the PureGene Extraction kit (Gentra Systems, Inc., Minneapolis, MN) according to the manufacturer’s protocol. PCR amplification with the L1 consensus primers Gp5+/Gp6+ gave an expected PCR product with approximately 140 bp. These primers allow the detection of a broad spectrum of mucosotropic HPV genotypes (6, 11, 13, 16, 18, 30-35, 39, 40, 42, 45, 51-53, 56, 58, 61, 66). However, no HPV DNA could be detected in the examined material.

**Figure 4:**
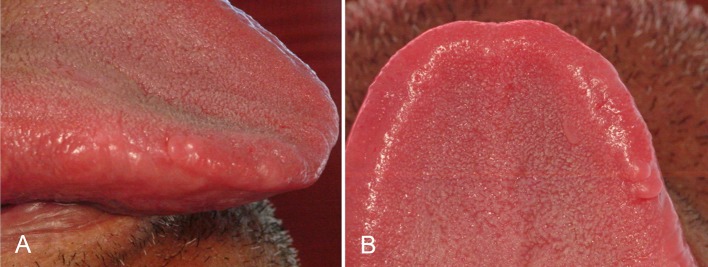
Clinical view, with no signs of recurrence after 36-month follow-up. Lateral view (A) and superior view (B).

The patient reported significant improvement in chewing after removal of the tongue nodule and there was no sign of recurrence after 36-month follow-up (Fig. 4). As the lip lesions did not cause any functional or esthetic discomfort, no surgical treatment was recommended, just reassurance and regular clinical monitoring.

## Discussion

FEH is a benign oral disease caused by HPV, especially 13 and 32 subtypes [6, 8, 12]. Although it is a rare condition, its diagnosis usually is not problematic, because the clinical presentation and demographic profile of the patients are quite characteristic [13]. Classically, FEH presents with multiple, normochromic, flat papules and nodules, located in the lips, buccal mucosa and tongue of children and adolescents, mainly those with indigenous heritage [10, 13]. In this paper, we described multiple mucosal lesions typically compatible with FEH; however, here they affected a 57-year-old Brazilian man, which is very uncommon finding. It is well documented that the great majority of FEH in the Americas is experienced by young patients [9-11, 13].

The occurrence of FEH in patients older than 45 years is extremely rare in the Americas. In 1994, Carlos and Sedano [9] reported 110 cases of FEH in patients living in Guatemala City and neighboring rural areas. These patients were aged between 5 and 38 years and 97% of them were in the first and second decades of life. Other authors from Central and South America also did not find Heck’s disease in patients older than 45 years [9-11, 13]. Then, this paper presented the first reported case of FEH affecting an older Brazilian adult, who was not self-declared as Indian. The epidemiological surveys performed by Correa et al [14] and Carvalho et al [15] did not report diagnosis of FEH in the Brazilian general population ≥ 60 years old after analysis of 2,250 and 543 biopsies, respectively. These studies corroborate the paucity of FEH in elderly patients in Brazil.

On the other hand, Axell et al [16] studied 20,333 adults in Sweden and found a prevalence of FEH of 0.11%, the disease being most prevalent in age groups above 45 years. Ratifying the presence of FEH in adults, Henke et al [17] described 16 cases of FEH in Germans aged between 22 and 85 years. In addition, Clausen et al [18] had reported FEH in Greenland Eskimos, both children and adults, ranging in age from 2 to 79 years. Likewise, scarce case reports of FEH in patients over 60 years have been reported in Germany [5], Sri Lanka [6] and India [19].

As there are many indigenous groups in Brazil and there is great miscegenation, more FEH reports among adults and the elderly in the Brazilian population would be expected. Indeed, the patient reported here may have unknown or denied indigenous descent, since he is from northern Brazil where there are many Indian tribes. It is noteworthy that Indian heritage is still regarded with a certain prejudice in Brazil. Factors that could explain the lack of FEH reports in adults and the elderly in Brazil include a particular tendency to spontaneous regression and the difficulty of access of poor people to health services, since FEH usually affects individuals who live in poverty or are of low socioeconomic status [8, 9, 11]. Further studies are warranted to elucidate such statements.

The identification of Heck’s disease is strongly suggested by the clinical findings [20], but histopathological analysis is usually required to confirm the precise diagnosis [8]. In some instances, condyloma acuminatum, verruca vulgaris and other rare conditions must be considered in the differential diagnosis and then to impose the necessity of microscopic examination [6-8]. The histopathological scenario of our case showed parakeratosis, acanthosis, rete pegs with a club-shaped appearance and two cytopathological features very characteristic of HPV infection, the presence of koilocytosis and mitosoid cells, a pathognomonic sign of FEH. In fact, FEH can be microscopically differentiated from viral warts by the presence of mitosoid bodies [6, 7]. Those microscopic findings in conjunction with clinical presentation were sufficient to establish the final diagnosis of FEH.

Although PCR analysis is a highly specific method and considered the gold standard for the detection of HPV [8, 21], our PCR investigation has failed in identifying the presumed HPV subtype in the excised lesion. This could be due to deterioration of DNA structure in paraffin-embedded block after a long time [22]. In fact, our PCR study was performed 6 months after the histopathological examination. Lack of identification of HPV by PCR in histopathologically confirmed cases of FEH was also reported by other authors [6, 23].

Treatment of FEH is not always required since the lesions are asymptomatic and may undergo spontaneous remission and there is no tendency to malignant transformation [8, 24]. We performed the excisional biopsy of the solitary tongue nodule based on patient’s complaint of persistent biting of the lesion during mastication. It was producing bleeding and intense masticatory discomfort. Indorsing this statement, Carlos and Sedano [9] showed that of the 110 patients studied, 49% reported that FEH lesions interfere with chewing and were accidentally bitten. In our case, a biopsy also allowed the microscopic diagnosis. Lesions that do not remit and cause functional and/or esthetic concerns may be managed by several means, including scalpel surgery, cryotherapy, CO_2_ laser, electrocoagulation, chemical agents (e.g., retinoic acid, imiquimod) and immunostimulants (e.g., interferon) [8, 24].

In conclusion, FEH is a rare benign disease of the oral mucosa associated with HPV infection. The disease is usually diagnosed in young people of indigenous heritage, but other ethnic groups and older patients may be affected. Herein, we reported a case of FEH in a 57-year-old Brazilian male, presenting with extensive lesions in the lips, buccal mucosa and tongue. Hence, clinicians must be aware of such rare presentations and accomplish a prompt diagnosis and appropriate treatment according to esthetic and functional purposes.
